# Janus-Nanojet as an efficient asymmetric photothermal source

**DOI:** 10.1038/s41598-022-17630-0

**Published:** 2022-08-20

**Authors:** Javier González-Colsa, Alfredo Franco, Fernando Bresme, Fernando Moreno, Pablo Albella

**Affiliations:** 1 Group of Optics, Department of Applied Physics, University of Cantabria, 39005 Santander, Spain; 2 Department of Chemistry, Molecular Sciences Research Hub, Imperial College London, London, W12 0BZ UK

**Keywords:** Nanophotonics and plasmonics, Nanophotonics and plasmonics

## Abstract

The combination of materials with radically different physical properties in the same nanostructure gives rise to the so-called Janus effects, allowing phenomena of a contrasting nature to occur in the same architecture. Interesting advantages can be taken from a thermal Janus effect for photoinduced hyperthermia cancer therapies. Such therapies have limitations associated to the heating control in terms of temperature stability and energy management. Single-material plasmonic nanoheaters have been widely used for cancer therapies, however, they are highly homogeneous sources that heat the surrounding biological medium isotropically, thus equally affecting cancerous and healthy cells. Here, we propose a prototype of a Janus-Nanojet heating unit based on toroidal shaped plasmonic nanoparticles able to efficiently generate and release local heat directionally under typical unpolarized illumination. Based on thermoplasmonic numerical calculations, we demonstrate that these Janus-based nanoheaters possess superior photothermal conversion features (up to $\Delta T\approx 35$ K) and unique directional heating capacity, being able to channel up over 90% of the total thermal energy onto a target. We discuss the relevance of these innovative nanoheaters in thermoplasmonics, and hyperthermia cancer therapies, which motivate the development of fabrication techniques for nanomaterials.

## Introduction

Cancer is one of the major causes of mortality worldwide^1^. Curing cancer is a key priority to society. Scientists have developed a variety of approaches to fight cancer^2^. Radiation therapy, chemotherapy or surgery are regarded as the main clinical treatments, but they suffer from many limitations, such as surgical complications and the serious side effects associated to radio and chemotherapy. Moreover, the efficacy of these three treatments is masked by metastasis phenomena^3^ which render cancer cells hard to be completely eradicated, leading to a poor patient survival rate. Thus, novel side-effect free therapies with higher effectivity are pressingly sought as an alternative to treat cancer patients. Within the alternative treatments, localized hyperthermia has flourished as a remarkable curative modality in which thermal agents are used to transfer heat to cancer cells, promoting their necrosis^4,5^. In particular, nanomaterials-based Photothermal Therapy (PTT) is appealing, since the ability to generate highly focused temperature spots diminishes the damage of surrounding healthy tissues. PTT relies on nanomaterials as photothermal agents and near infrared (NIR) laser irradiation to increase their temperature. Unlike conventional cancer therapies, PTT is a minimally invasive technique that may potentially allow a fast patient recovery with controllable, high selective and favourable biosafety features^6,7^. One of the main difficulties in the implementation of PTT, is the necessity to deliver a high amount of energy to the cancer cells, often located deep into the tissue. However, PPT can be combined with other therapeutical strategies such as surgery or chemotherapy^8–12^, thus increasing their efficacy, and overcoming the weaknesses.

The effectiveness of PTT often comes from the efficiency of the local heating source, i.e., its optical absorption and its biofunctionalization^13^, which is required to recognize the carcinogenic cells, for selective targeting. Various types of photothermal agents have been investigated for PTT applications, including bovine serum albumin heterojunctions^14^, graphene-based nanostructures^15^, but the PTT agents based on Localized Surface Plasmon (LSP) resonances are more widespread and promising^16^. These nanoheaters generate heat via resistive losses associated to light absorption at the plasma eigenfrequency. The absorption wavelength is very sensitive to the nanoparticle shape, size and composition^17–19^, hence allowing the spectral tunability of the heating. The ability to control heating at the nanoscale has opened a new research area^20^, thermoplasmonics, which encompasses most modern applications of nanoscale heating using plasmonic effects, including PTT. Gold and platinum are widely extended materials in PTT applications, due to their compatibility with biological systems and chemical inactivity. Most noble metals usually present LSP resonances in the UV–Vis range. In the cases of gold and platinum, their plasmonic resonances can be shifted to the first NIR biowindow (NIR-I, 700–900 nm)^21^, where light/heat conversion can be maximized due to a minimization of light absorption by water. Photothermal agents in the so-called second NIR biowindow (NIR-II, 1000–1400 nm) offer advantages in clinical applications due to the deeper penetration depth and lower optical absorption compared with NIR-I. Up to now, most of the LSP-based photothermal agents applied for PTT are mainly restricted to single-metal nanoheaters including gold nanospheres^22–25^, nanorods^26^, nanostars^20,27^ or nanodisks^28^. In fact, we have recently compared the thermoplasmonic behaviour of these geometries against doughnut-shaped nanoheaters, showing that the latter feature better thermal performance for photothermal applications^29^. Despite its high heating efficiency, these single-metal nanostructures heat up the surrounding environment isotropically leading to a partially uncontrollable heating effect. This feature does not only carry the potential damage of the targeted cancerous cells, but also the undesirable damage of the neighboring healthy cells. This is one of the challenges we need to surpass for a successful incorporation of PTT in clinical treatments. One important challenge in this context is controlling the heating approach, as substantial heating often requires collective nanoparticle effects to surpass the limitations of the incident power density. As a matter of fact, the Maximum Permissible Exposure (MPE) of skin is 1 W/cm^2^ in the NIR-II^30^), which would result in a modest temperature increment at single particle level. However, the operation intensity can be optimized by enhancing the light/heat conversion to maximize the energy output at the single particle level. Fortunately, the development of novel fabrication methods has provided feasible resources to overcoming this issue, offering novel structures with a superior thermo-optical performance, mainly based on the use of hybrid nanomaterials^31–33^.

Janus Nanoparticles (JNPs) are nanostructures composed by separated regions of different chemical compositions. They have attracted great attention in the area of nanomedicine^34–39^ because the combination of materials with different chemical and physical properties yields unique dual-functional capabilities offering new opportunities in fields such as imaging or drug delivery^40^ for biomedical applications. Colloidal JNPs have also been eventually explored to enhance the thermal performance of heating agents, such as multifunctional ternary JNPs^30^, UFO like hybrid cyclodextrin-Pd nanosheets^41^, silver-silica nanoplatforms^42^, gold triangle-mesoporous silica structures^43^, hybrid gold nanostars^44,45^ or different core–shell geometries^46–49^. So far, although the application of multifunctional hybrid particles is growing rapidly in nanomedicine, the design of highly efficient single JNPs with exceptional directional photothermal conversion abilities is still missing. Nanoheaters commonly show a remarkable symmetrical temperature distribution (around the main axes of the nanoparticle for anisotropic geometries) hence heating the environment around them homogeneously. To enable heating in specific directions, alternative nanostructures must be designed.

In this work, we present an exhaustive numerical thermoplasmonic study of a novel and effective prototype of a Janus heating nanojet (called J-Nanojet from now on) that offers improved photothermal conversion features together with remarkable directional heating capacity, thus, overcoming the aforementioned main challenge in PTT: reduce healthy tissue damage. The nanostructure proposed in this work is capable to channel up to 91% of the total heat in one direction, i.e., towards the cancerous tissue. The J-Nanojet is made by a metallic toroidal nanoheater unit embedded in a Janus capsule built of two different biocompatible materials, one acting as heat insulator and the other as heat conductor. We propose the use of fully biocompatible plasmonic materials such as gold or platinum^50^ as heating agents to tune the working wavelength of this J-Nanojet from NIR-I to NIR-II depending on the specific needs of applications. As it is usual in personalized cancer treatments such as in plasmonic photothermal therapies, the nanoparticles acting as nanoheaters are biofunctionalized to increase their selectivity towards specific targets^51^. In our theoretical proof of concept, we also propose that the J-Nanojet should be functionalized along the highly thermally conductive surface only. This would allow for a directional local heat delivery towards the cancer tissue preventing or reducing the damage of the healthy one. Finally, we numerically tested the heating performance of the J-Nanojet under dynamic flow conditions, i.e. considering the thermal behaviour stability to J-Nanojet random rotations when illuminated with unpolarized light, as expected to happen in a practical situation. We introduce here an accurate numerical model to test the performance of the proposed nanostructures. We demonstrate that the combination of metallic nanoparticles with diamond or PDMS provides a route to optimize the heat transferred from the nanoparticles to the medium, as well as ensuring optimal light absorption in the relevant 1st and 2nd biological windows. Hence, we believe that our work will motivate the fabrication of JNPs, opening a route to incorporate these nanostructures in photothermal therapies.

## Methods

Noble metal nanoparticles are highly effective heat generators when correctly illuminated. If light interacts with metallic nanoparticles, their free electron density starts to oscillate following the incident electric field, heating the metal via Joule effect. Then, the structure acts as a thermal source, heating up its surroundings. This process is commonly described by the well-known heat equation in spherical coordinates:

1 $$ \rho \left( r \right)c\left( r \right)\partial T\left( {r,t} \right)/\partial t = \nabla\,\cdot \,(k\left( r \right)\nabla T\left( {r,t}) \right) + Q\left( {r,t} \right) $$

where the variables r and t are the radial position and time, *T*(*r*,*t*) is the local temperature and the material parameters *ρ*(*r*), *c*(*r*) and *k*(*r*) are the mass density, specific heat and thermal conductivity, respectively. *Q*(*r*,*t*) is the heating source determined by the resistive losses in the metal. Therefore, for a fully characterized system (well-known nanoparticle and surrounding thermo-optical properties), the electromagnetic problem must be solved to obtain these losses. To do so, the system of Maxwell’s equations, and subsequently, the heat equation with appropriate boundary conditions have been solved by means of finite element simulations. For convenience and reliability of the solution, we have used Comsol Multiphysics 5.6, which provides with robust and solid numerical methods to solve partial differential equations. In our optical modelling, particles are supposed to be immersed in water and illuminated with circularly polarized light instead of unpolarized. Although this last polarization state could be simulated by just solving two different situations with orthogonal incident electric fields, this would double the simulation time. Thus, circular illumination is computationally more efficient, allowing the solution of the problem in just one step. We have considered the electromagnetic losses as the only heat source and the thermo-optical properties have been taken from Comsol database. A heat flux boundary condition has been imposed at the outer simulation frontiers together with a heat transfer coefficient to include the heat dissipation in our modelling. Therefore, we estimated the heat transfer coefficient by simply dividing the surrounding fluid thermal conductivity by the simulation region length scale.

## Results

### First steps: Hybrid nanoparticle designs

**Figure 1:**
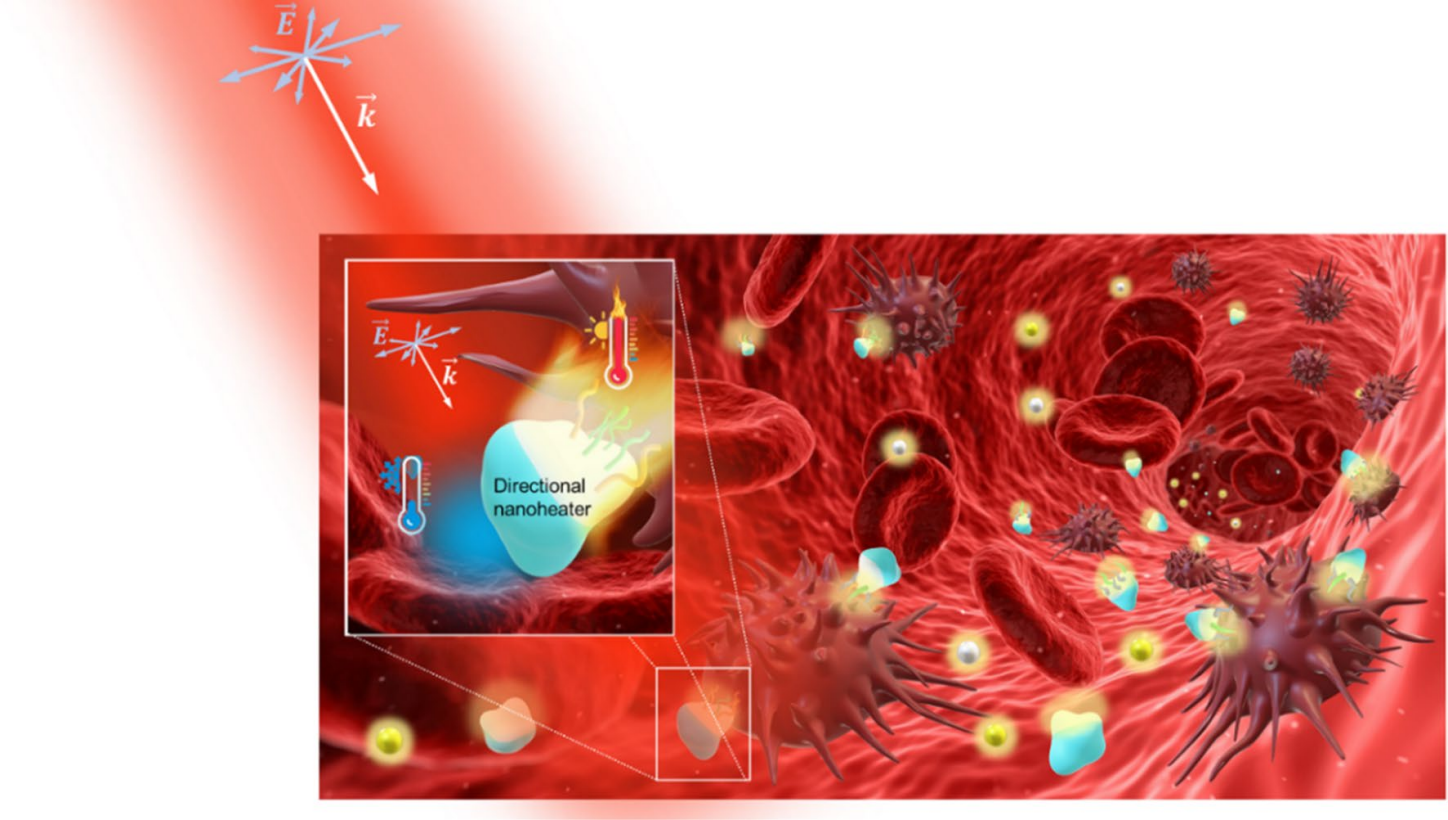
Illustration of spherical nanoparticles and abstract hybrid nanonheaters flowing inside a blood vessel in colloid under infrared laser beam illumination. Inset: abstract J-Nanojet structure reacting to the incident light, heating up a target cancerous cell. Feature Background Image Credit: “Blood cells” by qimono. CC0 Public Domain via Pixabay.

Most experiments focused on photothermal biomedical applications consider specifically biofunctionalized metallic nanoparticles in a fluid, which eventually binds to the target cells^52^. These nanoparticles, under an adequate illumination, become an LSPR-assisted heating source. In the specific case of PTT against cancer, the local temperature of the tissues must reach 42–48 degrees centigrade^7,20^, a temperature increment of approximately 10 °C above the average temperature of the biological medium. However, a precise control of the temperature increases is required to avoid uncontrollably damage of the healthy surrounding tissue, as it would happen with a continuous wave laser. Tissues can be affected in different ways: photochemical damage, photo-thermal damage, photoablation or photodisruption, depending on the power density and the light exposure time^53^. Pulsed lasers are widely used to control the energy delivery circumventing these issues, allowing high thermal responses at low power densities. In the context of photothermal therapies, this could be achieved by designing more efficient single nanoheaters by optimizing light/heat conversion and energy delivery (local directionality). Specifically, PTT also requires the delivery of optical energy to a target sometimes localized deep into the tissues. This can be achieved by illuminating at the right biological window (NIR I-II) where the tissue penetration depth can be few microns up to 10 cm^53–55^. As illustrated in Fig. 1, the temperature spatial distribution generated by single nanoparticles immersed in a fluid has strong symmetries, as is the case of the gold nanospheres, resulting in a radial stationary isotropic thermal flux. This spatial uniformity leads to a dramatic reduction in the heat transfer control since the thermal energy flows in all directions, i.e. only a portion of the generated heat reaches the target cells, with the rest of the heat diffusing towards surrounding tissues. Consequently, a great part of the available thermal energy is wasted.

In light of this, to increase the heating efficiency and to maintain its biosafety, it is vital to improve the thermal capabilities of the nanoheaters in terms of temperature generation but more important, in thermal flow control. A clear step forward from the existing single-material structures is to consider asymmetric nanoparticles made of two different materials, a typical JNP, where each half of the particle volume is occupied by one of them, we call this a “hybrid nanoheater”. Both materials must be selected with radically opposite thermal conductivities. We propose a hybrid nanoheater composed by polydimethylsiloxane (PDMS) and gold or platinum, acting the first one as an efficient heat insulator and the second one as the heating source.

**Figure 2:**
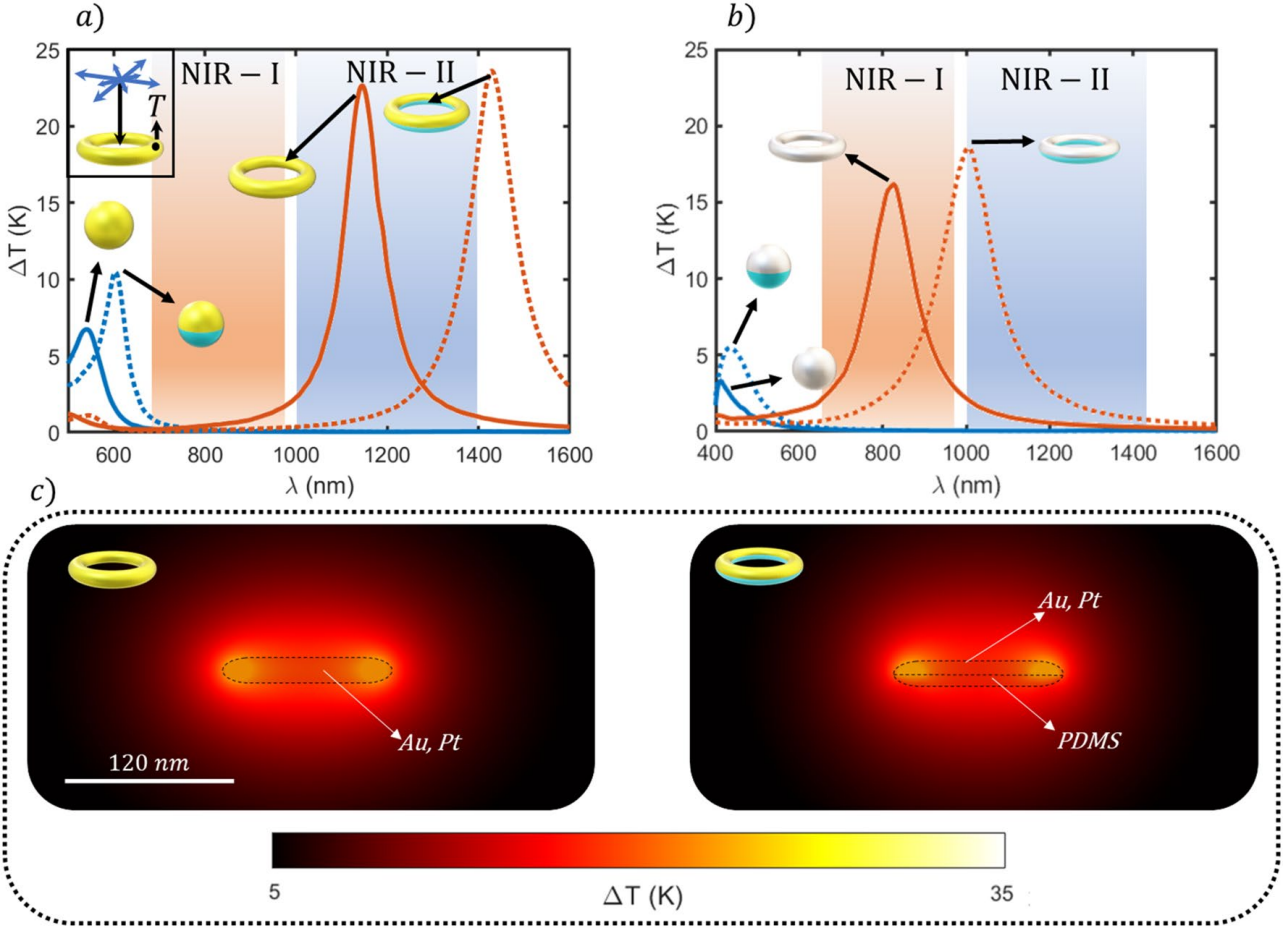
Thermal spectrum comparison of the studied single-material and hybrid nanoheaters for gold (**a**) and platinum (**b**) irradiated with a power density of 0.1 mW/μm^2^; solid and dotted lines correspond to single material and hybrid nanostructures respectively. The reddish and blue shadowed regions represent the first and second biological windows (NIR I-II) respectively. The temperature is taken at the point *T*, inside the metal material for all structures. (**c**) Temperature maps for the optimal wavelength in (a) for the single material and hybrid toroids immersed in water.

Figure 2 shows a comparison of the heating spectral response of a single material nanoparticle with a hybrid Janus one. Two different shapes are considered: sphere and toroid. In Fig. 2a the particles are made of gold and in (b) made of platinum. The dimensions of the nanoparticles acting as nanoheater units were selected from the optimal ones reported in^29^: the optimal sphere has a radius of 40 nm while the toroid has a 50 nm main radius and 10 nm secondary radius.

In the single-material sphere case, the maximum achievable temperature increment (ΔT) increases when the isolating material is added for both, Au and Pt, and their thermal response is also redshifted, approaching the NIR-I. However, both maxima show a poor temperature increment for the fixed incident power density (0.1 mW/μm^2^) and are located out of the NIR biowindow, becoming inadequate for biomedical applications. On the other hand, the toroidal-shaped nanoheaters display superior heating performances with respect to the spherical cases. It is noteworthy the effect of the particle materials.

The spectrum of the Au toroid lies out of the NIR-II when PDMS is added. Although that response can be tuned back to the NIR-II by reducing the dimensions of the toroid, the maximum ΔT is also decreased (see Figure S1 of the supplementary material). This dramatically reduces its heating potential and consequently, the applicability in biomedical applications. The Pt toroid spectrum is also redshifted with respect to the single-material nanoheater, remaining at the threshold of the NIR-II. Despite this hybrid nanoparticle offers a favorable temperature increase, particles able to respond to excitation wavelengths further in the NIR-II are more desirable since the tissue penetration length is larger in that region. In Fig. 2c the temperature maps of single material and hybrid nanodoughnuts are shown. It can be clearly seen that both architectures exhibit a similar temperature distribution, but the hybrid one is slightly asymmetrical. Thus, the typical Janus nanoparticle, called here hybrid nanoheater, presents a low degree of anisotropy, suggesting a poor achievable directional transfer. These temperature distributions remark the necessity of improving the nanoheater thermal directivity while preserving its power generation efficiency. However, although some insights can be obtained from the temperature spatial distribution to estimate the thermal directional capabilities of nanoparticles, it is necessary to quantify their anisotropy degree to select the best candidate depending on the specific biological application. This can be achieved by calculating the resulting thermal flux in a fluid. We assume that radiation heating can be neglected regarding the magnitude of the temperatures we are considering (biological medium temperature).

**Figure 3:**
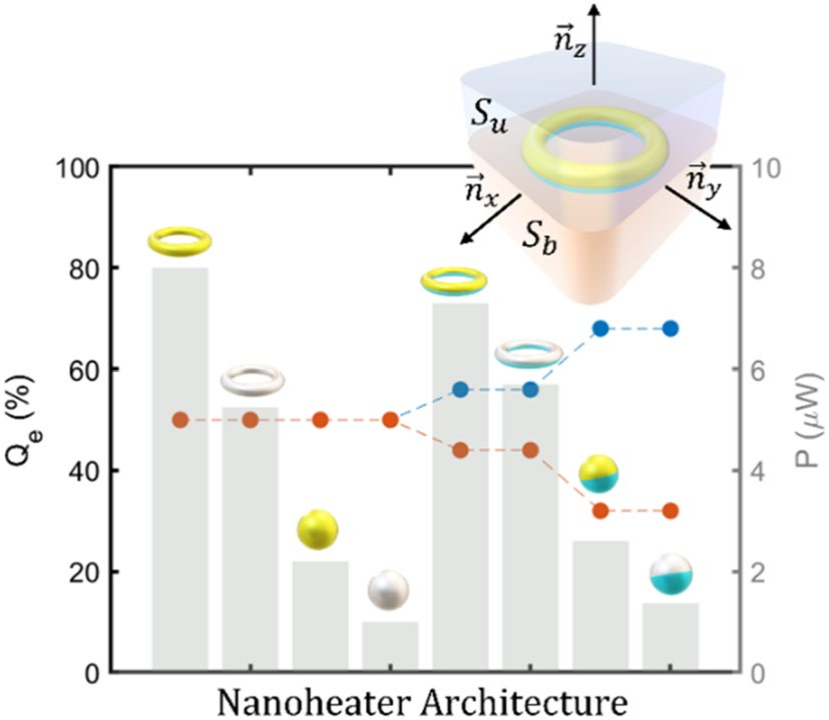
Comparative of the relative conductive heat power (*Q*_*e*_) flowing through the upper half-space (in blue) and lower half-space (in orange) for single material and hybrid nanoparticles irradiated with a power density of 0.1 mW/μm^2^ at the resonance. The grey bars correspond to the total heating power for each architecture. Inset: Scheme of the conductive thermal power calculation method applied to the nanoheaters where *S*_*u*_ y *S*_*b*_ are the upper and bottom half-spaces respectively. *n*_*x*_, *n*_*y*_ and *n*_*z*_ are the canonical basis vectors.

The convective heating can also be neglected since surrounding fluid is supposed to be stationary, being the system analysed only half micron far from the nanoheater. Therefore, we are considering that the heat transfer in a complex biological fluid is expected to be mainly owing to conduction^6^. To properly characterize the directional capabilities of nanoheaters, i.e., the ability to drive directionally the heating power, two magnitudes must be analyzed: the relative power flowing through the upper and bottom half-spaces (*Q*_*e*_) and the total generated power (*P*). The followed strategy to calculate these magnitudes is illustrated in the inset of Fig. 3. A cubic surface surrounding the nanoheater and centered at its geometrical center has been considered. This geometry has been selected for convenience in the data management of the simulation software (Comsol Multiphysics). Then, the temperature gradient is integrated along the upper (*S*_*u*_) and bottom (*S*_*b*_) half-spaces (integration domain) to obtain the ratio of heating power going through them.

The results of the estimated relative heat power for each nanoheater flowing through the upper media are shown in Fig. 3. It can be seen that spherical nanostructures reach a maximum value of 68%, while toroidal nanostructures offer 56%, in contrast with the single-material particles which as expected, are totally symmetric displaying a relative integrated flux of 50%. Nevertheless, although hybrid spheres display higher directional capabilities, they generate the lowest thermal powers when compared to toroidal geometries. Therefore, the hybrid nanoheater geometries clearly show an improved heat directional transfer with respect to the single-material structures while keeping the achievable maximum temperature (Fig. 2) and the generated total power (Fig. 3) almost constant. Even though this is already an interesting result, we take this one step further to magnify both, the directional capabilities and the total generated heating power for each architecture.

### J-Nanojet design: Thermal response optimization

**Figure 4:**
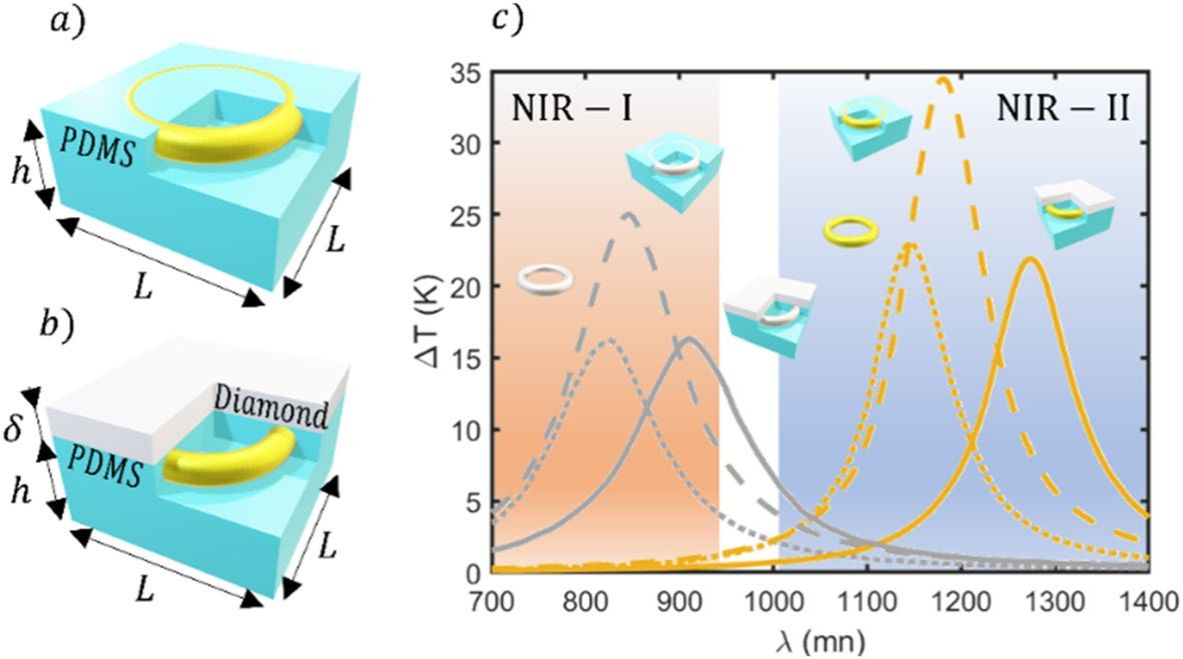
Illustration of the J-Nanojet architectures: a metallic toroid embedded in a PDMS block (**a**), covered with a thin squared diamond layer (**b**). An illustrative section has been made in the nanoparticles to visualize the metallic heating unit. The variables *h*, *δ* and *L* represent the capsule parameters: PDMS thickness, diamond thickness and the size of the capsule base respectively. (**c**) Thermal spectrum comparison of the structures shown in (**a**) and (**b**): an isolated metallic toroid (dotted line), a toroid embedded in a 125 × 125﻿ × 50 nm block of PDMS (dashed line) and the ternary J-Nanojet structure (solid line). The blue and reddish shadowed regions represent the first and second biological windows respectively.

In sight of the previous results, designs that allow to enhance the heating performance asymmetry while improving the maximum achievable temperature and power are sought. To achieve this aim, we have designed and optimized nanostructures, “heating nanojets”, which provide heating in one direction, reducing the heating of the opposite direction. The illustrations in Fig. 4a–b, show the proposed basic scheme of the two different J-Nanojet designs, so-called binary and ternary. While the ternary structure allows for a slightly better control of the local heating, it is more challenging to achieve from the fabrication point of view. Conversely, the secondary architecture can be manufactured using state of art fabrication techniques^56^. We have considered a cubic geometry to facilitate the numerical modelling of the thermoplasmonic response. We note however that the ideas presented here apply to other geometries too, which incidentally are less demanding from the fabrication point of view (see Figure S2 in the supplementary material). The first design (Fig. 4a) consists of a doughnut-shaped metallic nanoparticle embedded in a Polydimethylsiloxane (PDMS) capsule (binary J-Nanojet), touching the upper boundary of the polymer in contact with the surrounding fluid. The second one (shown in Fig. 4b), is an extension of the binary nanojet, by just adding a thin diamond cap on top of the toroid to force the heat to flow in the upper direction (ternary J-Nanojet). PDMS is an effective thermal insulator and diamond a good thermal conductor, with thermal conductivities of ~ 0.15 W/mK^57^ and 2200 W/mK^58^, respectively. Furthermore, considering its good thermal performance, the optimal toroid from^29^ has been selected as the heating unit. This selection guarantees the maximum achievable temperature increase for excitation wavelengths in the NIR biowindow. This is essential in real PTT applications where nanostructures are randomly oriented and human tissues induce a partial depolarization of light with an exponential decay of its intensity. Hence, the J-Nanojets are always analyzed under unpolarized illumination with a pumping power density of 0.1 mW/µm^2^. Furthermore, in PTT, nanoheaters would circulate through thin capillaries, thus, the size of the agent acting as nanoheater must be controlled and below a certain allowed value. This condition has been satisfied considering nanojet designs with all dimensions around a therapeutically allowed of 150 nm^17^.

A previous optimization of the capsule geometrical parameters has been performed to visualize their effect in the J-Nanojet thermal response. Different values of the conductor and insulator thicknesses (*δ*, *h*) were considered, ranging from 10 to 30 nm and 20 to 50 nm, respectively (see Figure S3 in the supplementary material), reporting a notorious maximum temperature increase stabilization for δ = 10 nm and *h* ≥ 50 nm. The block base (*L*) of the nanojet has been considered to be 125 nm side to allow the torus to be fully embedded in the PDMS block while allowing for compactness.

Figure 4c), shows the results obtained after that optimization, comparing the J-Nanojet designs with the isolated heating unit. This allows to select the best design in terms of potential heating capability, i.e., maximum temperature increase and spectral location of the thermal response. Notice also that, although similar trends are found for both heating unit materials (Au and Pt) in both J-Nanojets (binary and ternary), the platinum thermal response is broader, thermally weaker, and its effective response is located at the NIR-I. On the other hand, the gold core gives higher temperatures but at the NIR-II. This fact opens the possibility to extend the use of this nanostructure to other biomedical applications, where the tissue penetration length is not a critical parameter and gives the platinum structure more tolerance to fabrication imperfections. However, it can be clearly seen that in terms of the maximum temperature achievable, the binary design outperforms the ternary one, reaching increments of 53–58% while equivalent values are obtained compared with the isolated doughnut (see Figure S4 to visualize the thermal response of the ternary design covered with sapphire as an alternative to diamond). For a better understanding of the J-Nanojet thermal response and to show why they can suppose a significant advance in certain photothermal applications, such as PTT, the previous results are complemented with their spatial temperature distributions.

**Figure 5:**
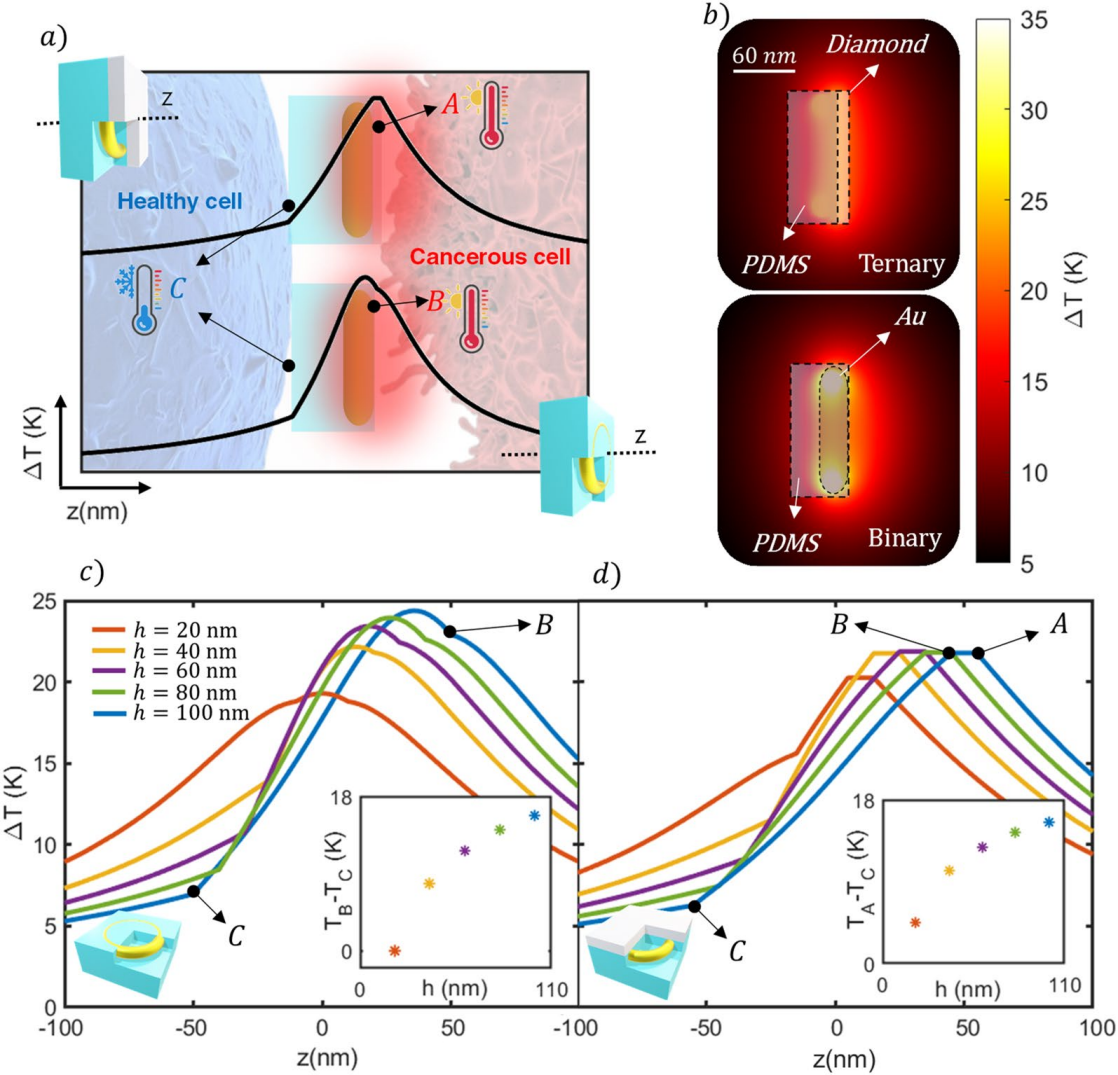
(**a**) Illustration of the typical thermal profile taken along the z axis for the J-Nanojet architectures where A and B are the PDMS boundaries, and C is the boundary of the diamond. (**b**) Temperature spatial distribution of the different J-Nanojets in water excited at λ = 1180 nm (binary) and λ = 1275 nm (ternary). Comparison of thermal profiles along the z-axis for a set of insulator thicknesses ranging from 20 to 100 nm in 20 nm steps for the binary (**c**) and ternary (**d**) J-Nanojets.

Before analyzing the spatial thermal distribution that these nanojets can offer, an illustration of the working principle is shown in Fig. 5a). As can be seen, the heat flows preferentially through the material with higher thermal conductivity, while PDMS introduces a resistance to heat flow. The heterogeneous structure of the material induces a noticeable asymmetry in the temperature profiles along the *z* axis (binary nanojet), which can be enhanced by adding a thin diamond layer (ternary nanojet). Thus, the asymmetrical *z*-profiles suggest that both architectures may present a high temperature contrast between the front (A for ternary and B for binary J-Nanojets) and rear boundaries (C). This induced thermal Janus effect, enables the possibility of affecting the target cells while protecting the rest of healthy tissue.

Figure 5b shows the temperature maps for the optimal binary and ternary J-nanoheaters, where the aforementioned thermal distribution asymmetry is demonstrated. Although the binary nanojet presents the highest temperature increase, the corresponding temperature map evidences how this temperature is confined inside thermal insulator, resulting in heat inefficiently transmitted to the surrounding fluid. Furthermore, both J-Nanojets show comparable spatial thermal distributions, that tend to be almost isotropic at large distances from the heating device. However, in the case of PTT, the tumor cells would be located at a distance of around 10–30 nm from the J-Nanojet surface due to the biofuntionalization agents^59^ (see Fig. 1), so that the short distance heating performance needs to be analyzed in detail. Moreover, one of the most important aspects to consider is the precision in the thermal control.

In photothermal applications, the temperature increase is sought to be stable and controllable, so temperature gradient-free structures are desirable since they allow for a high temperature decay control along a great surrounding volume. In order to get deeper insights of an optimal thermal agent prototype, it is necessary to analyze how the temperature decays with respect to the distance from the front and back nanoheater surfaces. Figures 5c-d show the thermal profiles of the binary and ternary J-Nanojet excited at their most efficient wavelengths, 1180 nm and 1275 nm, respectively (extracted from Fig. 4c). It can be seen that the binary design shows higher temperature increments than the ternary, being comparable for thinner insulator layers. Equivalent results were obtained for the platinum heating unit (see Figure S5 in the supplementary document). An interesting situation appears for the 20 nm-thickness case, where the binary design offers a totally symmetric *z*-profile, leading to an absence of conductive thermal flow control, while the ternary nanojet presents an asymmetrical profile from the beginning, allowing a certain heat directionality. In that case, the temperature contrast between the front and the rear of the nanoheater side is converted from 0 to 4.6 K. Thus, the ternary nanojet architecture offers the possibility to partially propel forward the heat power, highly reducing backward heating, even for smaller insulator coverage which is favorable given the size limitations inherent to the biological medium of interest.

Also note that the maximum temperature reached along z-axis by both architectures evolves with the insulator size (see Figs. 5c–d). In the binary design case, this temperature increases as the PDMS is added, being stabilized for a thickness of around 100 nm. Conversely, the nanojet shows a remarkably stable temperature increment for a wide range of PDMS thicknesses, remaining its optimal thermal response intact facing fabrication imprecisions. Therefore, the nanojet structure may offer high tolerance to fabrication imprecisions being capable to direct heat for all thicknesses. On the other hand, the thermal response of the binary design is more sensitive to thickness variations becoming less tolerant to manufacturing irregularities. However, it presents a higher maximum temperature becoming more effective in light to heat conversion, so that, both structures offer an outstanding thermal performance regarding photothermal applications. A highly influential parameter to consider in the heating control optimization is the temperature gradient between the front and the rear of the nanoheater since it gives a clear idea about the nanoparticle ability to heat up only in forward direction, insulating backwards. The tendency of this magnitude is shown in the insets of Fig. 5c-d. For all cases, as the insulator thickness increases, the temperature contrast grows, leading to a profile asymmetry enlargement. Consequently, both architectures are more effective directing heat for higher insulator thicknesses, disregarding the core material.

### J-Nanojet directionality: Thermal conductive flux

**Figure 6:**
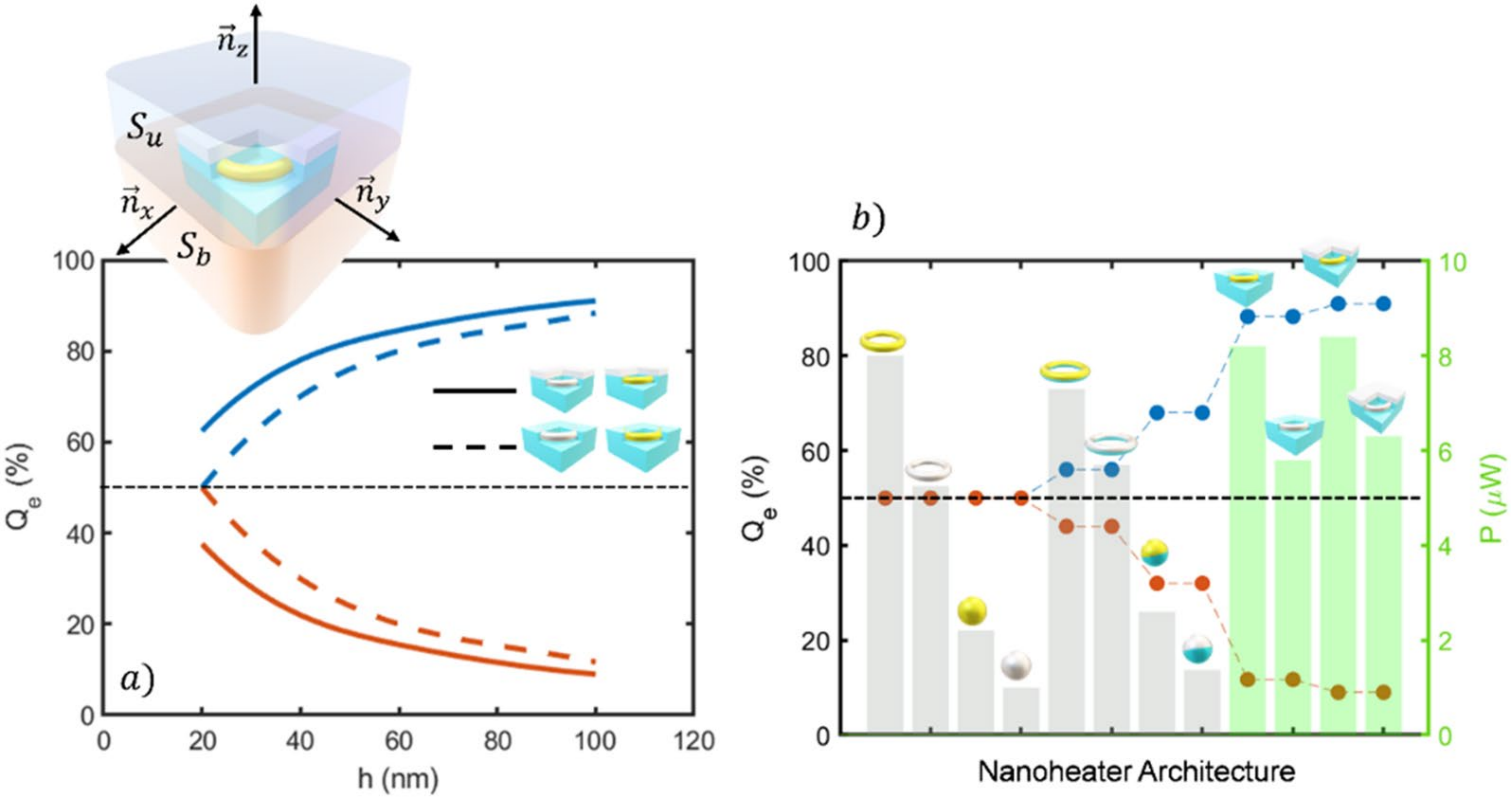
(**a**) Comparative of the relative conductive heat power (*Q*_*e*_) flowing through the upper half-space (in blue) and lower half-space (in orange) as a function of the insulator thickness h for gold and platinum heating units and both, binary (dashed line) and ternary nanojet (solid line) designs. Inset: Scheme of the conductive thermal power calculation method applied to the nanojets where *S*_u_ y *S*_b_ are the upper and bottom half-spaces respectively. *n*_x_, *n*_y_ and *n*_z_ are the vectors of the canonical basis. (**b**) Comparative of the maximum relative conductive power for all the considered structures: single-material/hybrid nanoheaters in grey and J-Nanojet designs in green.

In light of the previous results, the amount of heating power that J-Nanojets can provide and properly drive is calculated. The approach is similar to that followed for the hybrid structures. As illustrated in the inset of Fig. 6a, the heat flow through the faces of a cubic surface with the J-Nanojet at its center is analyzed. The results are shown in Fig. 6a, where the obtained heating power for binary and ternary J-Nanojets can be seen. One interesting feature that distinguishes both architectures is the relative heat power discrepancy for thin insulator thicknesses. As seen in the figure, the ternary J-Nanojet offers certain heating power control even for the thinnest PDMS covering.

Furthermore, as PDMS is added, the relative heating power directivity increases for both architectures since the insulator material inhibits heat transport through it while the diamond cap in the ternary nanojet promotes a flow in forward direction. Consequently, the efficacy of the binary and ternary nanojet structures are closer for larger PDMS sizes being able to direct forwards up to the 87% and 91% of the heating power respectively. These high directivities outperform the obtained for hybrid nanoparticles as illustrated in Fig. 6b, where a maxi-mum value of 68% is reached for spherical nanoheaters. The toroidal geometries offer a directivity of 56%, in contrast with the single-material particles which are totally isotropic. It must be noticed that under a 0.1 mW/μm^2^ power density illumination, spherical nanoparticles show the lowest generated thermal powers for all cases, becoming inferior compared with toroidal geometries. In contrast, both J-Nanojets designs generate the highest thermal powers reaching more than 8 μW and 6 μW for gold and platinum cores, respectively. Thus, the J-Nanojet designs are capable of generate more heating power for similar input conditions resulting in more efficient architectures that are able to propel the greatest part of that energy forwards.

### J-Nanojet in fluid: Thermal dependance with orientation

As reported in^29,60^, another remarkable and highly influential aspect to consider in JNP-assisted biomedical applications is the fact that nanoparticles are immersed in a flowing free biological environment, it makes the nanoparticles to dynamically suffer rotations that strongly affect their photothermal conversion efficacy. Therefore, the effective temperature obtained in such a case is the average of temperatures achieved for all possible orientations with respect to the electric field of the incident light being more favorable those particles with a poor thermal dependence on the electric field orientation. Furthermore, biological tissues are expected to be absorbing and dispersive, properties that dramatically reduces the incident light degree of polarization.

**Figure 7:**
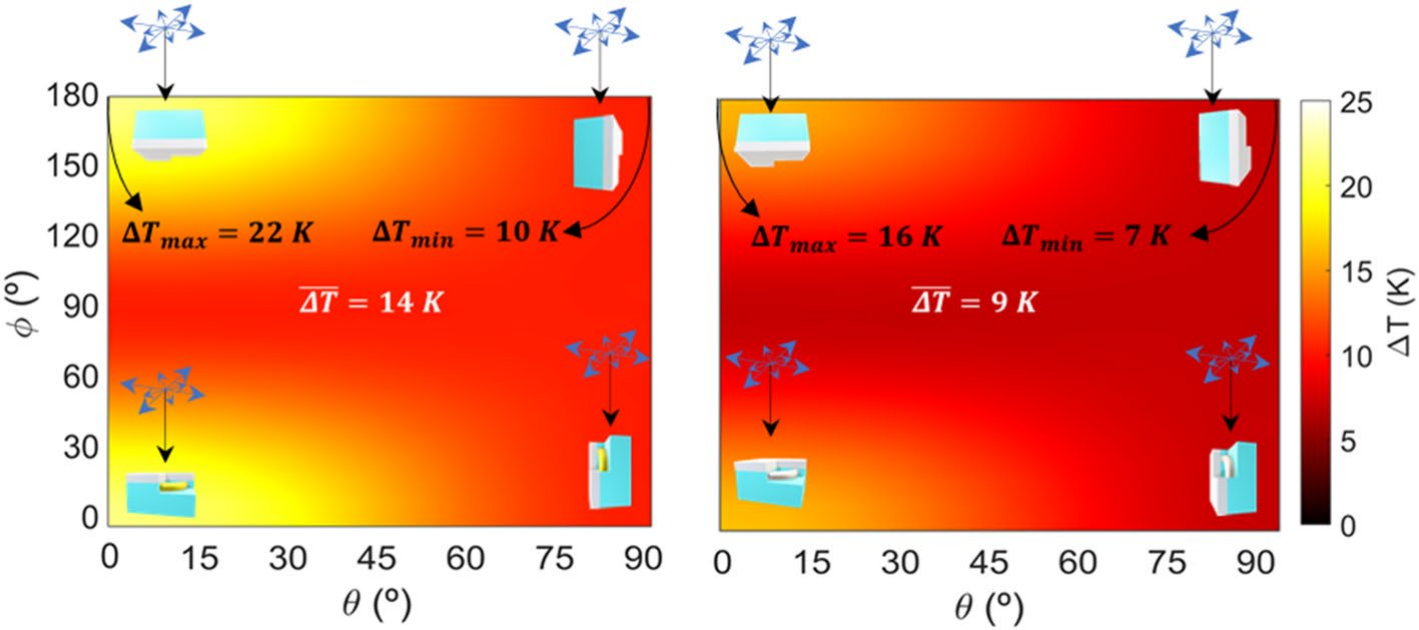
Thermal responses of the gold and platinum ternary nanojet to rotations. *θ* and *ϕ* are the rotations with respect to the *x* and *y* axes. The considered unpolarized light (insets) injects a power density of 0.1 mW/μm^2^.

To clearly discern the stability of the J-Nanojets under free rotations, we considered a ternary J-Nanojet immersed in water and calculated how their relative orientation to the incident light direction affects their thermal response. To do so, the thermal response of the ternary J-Nanojet was calculated for the optimal wavelengths taken from Fig. 4c for a set of 3D rotations equally weighted on the sphere^61^. Then, the numerical mean temperature increment is calculated. Figure 7 shows the temperature maps of the nanoparticles for all the studied 3D rotations with respect to the x and y axes, taking their optimal response orientation as the initial position. The system is analyzed for unpolarized light.

Attending to the Fig. 7, it can be clearly seen a revolution symmetry in the colormap, as the revolution axis of the nanojet and the *z* axis (beam direction) match in the initial configuration. Thus, considering the temporal evolution of the electric field vector for unpolarized light, rotating the nanojet about the y axis, and subsequently about the *x* axis, has the same effect as rotating it about the *x* axis and then about the *y* axis. To clarify this figure, setting *θ* = 0º and then increasing the rotation in *ϕ* will mean that eventually the transverse length of the toroidal metallic core and the electric field will be orthogonal, leading to occasional resonances due to alignments between this transverse length and the rotating electric field, resulting in a fall in temperature increment around 53% and 56% compared to the most favorable configurations (ΔT = 22 K and ΔT = 16 K) for gold and platinum, respectively. Thus, due to the nanodoughnut symmetry, the nanojet exhibits a more stable thermal response to rotation for both materials, offering average temperature increments of 14 K and 9 K, accordingly (a similar investigation for different capsule geometries can be found in Figure S2).

## Discussion

We have numerically investigated the photothermal behaviour of different novel Janus nanostructures, showing that a combination of materials with radically different thermal properties (PDMS/diamond) produces a strong thermal Janus effect allowing to control the heating direction and improving the efficiency of nanoheaters at the single particle level, which also boosts the collective thermal performance. The proposed doughnut-based architectures, the binary and the ternary nanojet designs, outperform simple Janus nanostructures such as the sphere and toroid geometries in terms of maximum temperature increase and thermal control. They reach temperature increments up to $\Delta T=35$ K and ${Q}_{e}=90\mathrm{\%}$ of thermal directivity in contrast with the hybrid toroid that shows $\Delta T=23$ K and ${Q}_{e}=56\mathrm{\%}$ of directivity under same illumination intensity. This makes the J-Nanojets a powerful tool in biomedical applications since it provides larger temperature areas supporting resonances within the therapeutic windows, also reducing the backward heating. On the one hand, the binary design is more sensitive to insulator thickness variations, however, it displays a superior maximum temperature increase and are potentially easier to fabricate. Meanwhile, although the hybrid nanojet structure offers a slightly weaker thermal response, it provides higher thermal control together with a remarkable tolerance to insulator thickness imperfections. We have also analyzed the effect that the hybrid nanojet orientation with respect to the source has on its photothermal performance. This structure features a high average temperature increment also showing a great tolerance to rotation which makes it a promising candidate for biomedical applications. We believe that the models proposed here provide a direction to manufacture efficient nanoparticles for PTT applications in a near future, and therefore we expect that our work will motivate experimental studies and the development of nanofabrication methodologies. In view of the proposed alternative mechanism to the actual photothermal techniques a natural step forward would be to extend the study to a more general system involving the ligand proteins that connect the nanojet and the cancerous cell to find its influence on the heat transfer to the cell.

## Supplementary Information

Supplementary Information.

## Data Availability

The authors declare that all data supporting the findings of this study are available from the corresponding author upon reasonable request.
