# Supplementary material for: Janus-Nanojet as an efficient asymmetric photothermal source
Source: Sci Rep. 2022 Aug 20;12:14222. doi: 10.1038/s41598-022-17630-0 (PMC9392775; doi:10.1038/s41598-022-17630-0)
Supplement: Supplementary file 1 — Supplementary Information. [file 41598_2022_17630_MOESM1_ESM.pdf]

Janus-Nanojet as an efficient asymmetric photothermal source

*Javier González Colsa[a], Alfredo Franco[a], Fernando Bresme[b], Fernando Moreno[a] and Pablo Albella[a]**

[a]Group of Optics, Department of Applied Physics, University of Cantabria, 39005, Santander, Spain.

[b]Department of Chemistry, Molecular Sciences Research Hub, Imperial College London, W12 0BZ, London, United Kingdom.

Email: pablo.albella@unican.es

SUPPORTING INFORMATION

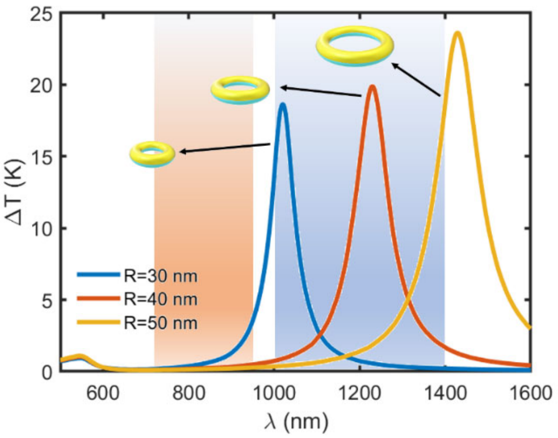

Figure S1. Thermal spectrum comparison of different Au/PDMS hybrid nanodoughnuts with 30, 40 and 50 nm main radii and 10 nm secondary radius. The reddish and blue areas determine the NIR-I and NIR-II respectively.

Figure S1 shows the thermal spectral response of hybrid nanodoughnuts composed by PDMS and gold for different main radii. As can be expected, when this parameter decreases the maximum temperature increment is reduced and so does the resonance wavelength. This leads to a compromise between size and thermal performance of nanoheaters. In this work, the best nanoparticle in terms of maximum temperature achievable has been considered, being selected the 50 nm main radius toroid.

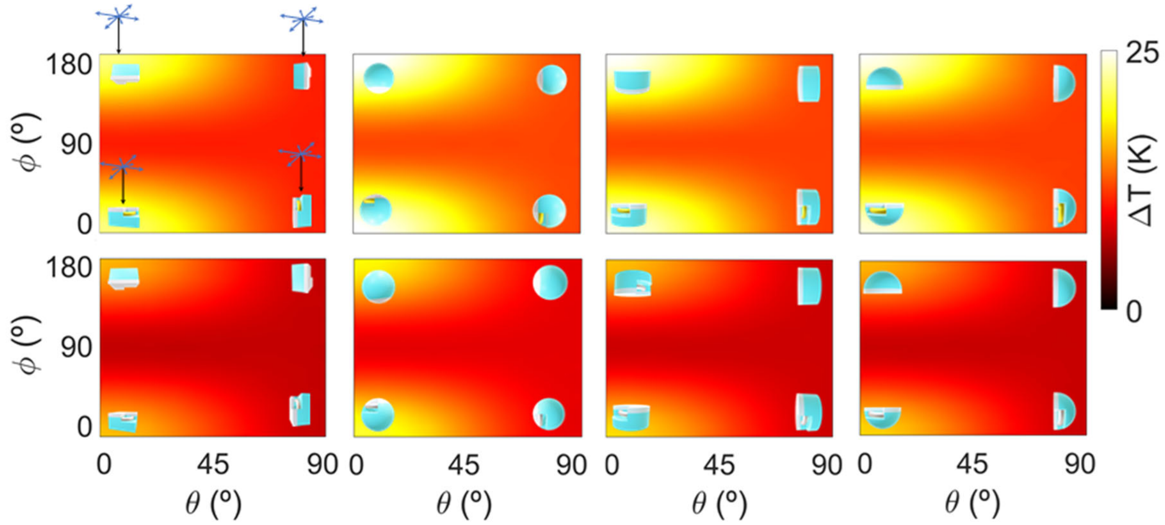

Figure S2. Comparison of the thermal response behaviour under rotations for different capsule geometries: rectangular (a, e), spherical (b, f), cylindrical (c, g), and hemispherical (d, h). First row plots correspond to the toroidal gold core and the second row to the platinum one. The incident beam was unpolarized for all cases.

Figure S2 shows the angular thermal response for different capsule geometries (rectangular, spherical, cylindrical and hemispherical). The particle rotations have been performed around two main axis, x and y, due to the usefulness of the FEM software. It can be clearly seen that all the capsule geometries offer equivalent angular responses for both core materials, gold and platinum, showing high stability under rotations in all cases. However, it is noticed that the spherical coverture presents a slight superiority with respect to the others.

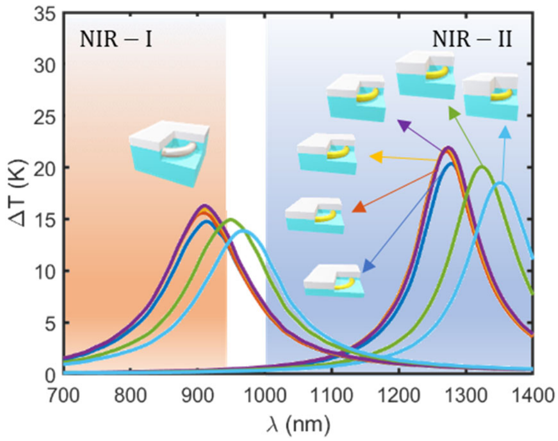

Figure S3. Capsule spectral thermal optimization for binary and ternary designs. The PDMS thickness varies from 20 to 100 nm and the diamond one from 10 to 30 nm.

Figure S3 shows the spectral thermal response of the ternary J-nanojet for different conductor/insulator thicknesses. The insulator thickness varies from 20 (which is required for the toroid to be fully embedded in the insulator block) to 100 nm and the conductor one from 10 to 30 nm. It can be seen the expected redshift of the platinum toroid response with respect to the gold case. In sight of the figure, the diamond cap has the major impact in the spectral location due to its high refringence. The maximum temperature is also decreased as diamond is added by cause of the thermal conductivity (2200 W/mK). Thus, the optimum spectral response is reached for insulator thicknesses between 50 and 100 nm and for a conductor thickness of 10nm.

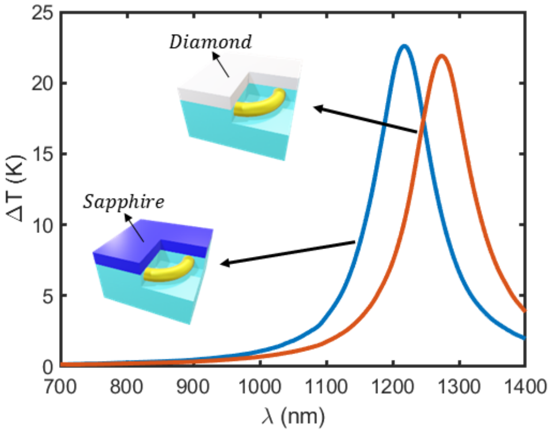

Figure S4. Comparison of the thermal spectrum for the nanojet structure covered with a 10 nm-thick layer of sapphire (blue) and diamond (red). The PDMS thickness is 100 nm.

Figure S4 plots the thermal response of two nanojets covered with a 10 nm-thick layer of sapphire and diamond respectively. It can be clearly seen how the diamond produces a spectral redshift with respect to the sapphire one. This can be analysed considering the refractive index values for both materials. In this spectral region, diamond presents a refractive index of around 2.38 RIU while sapphire possess 1.75 RIU. However, the most remarkable aspect is the slight discrepancy in the temperature increment. Despite the huge contrast of thermal conductivities (2200 W/mK for diamond and 35 W/mK for sapphire), the maximum temperature increase remains similar for both cover materials. This suggest that, disregarding the impact in the temperature increment, different materials with a wide range of thermal conductivities can be selected for the conductive cover layer, only considering other aspects such as the fabrication possibilities.

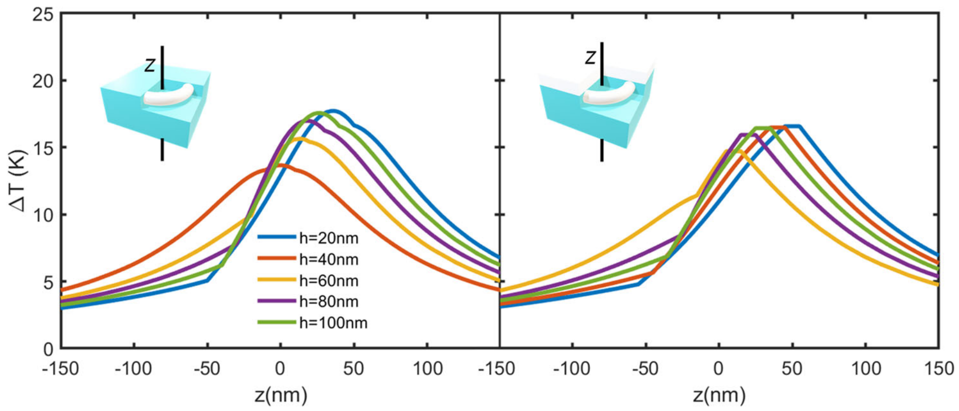

Figure S5. Extended comparison of thermal profiles along the z-axis shown in Figure 4 for a set of insulator thicknesses ranging from 20 to 100 nm for the platinum toroidal core.

Figure S5 shows the thermal profiles along the z-axis for the platinum core (binary and ternary J-nanojets) Accordingly to the figure 5 in the manuscript, it can be seen that the presence of a diamond layer in the ternary design induces a thermal stabilisation in its volume where the maximum temperature is reached. In contrast, in the binary design this maximum is reached inside the PDMS becoming useless. As in figure 5, paying attention to the thinnest PDMS capsule (*h*=20 nm), it can be seen that the ternary design offers certain asymmetry from the beginning while the binary does not. This can be favourable regarding the size limits required in biological media. Furthermore, the same behaviour of gold can be observed. As the PDMS thickness grows the maximum achievable temperature increases, being immediately stabilised in the ternary J-nanojet.
